# Screening and Cohorting of CRE Patients: The Strategic Role of Bed Management in a Monocentric Pre–Post Observational Study

**DOI:** 10.3390/antibiotics15030290

**Published:** 2026-03-12

**Authors:** Salvatore Altavilla, Nicoletta Di Pietro, Daniela Loconsole, Francesco Di Gennaro

**Affiliations:** 1 Azienza Ospedaliero Universitaria Consorziale Policlinico di Bari, 70124 Bari, Italy; 2 Clinic of Infectious Diseases, Department of Precision and Regenerative Medicine and Ionian Area (DiMePRe-J), University of Bari “Aldo Moro”, 70124 Bari, Italy

**Keywords:** bed management, infection prevention, carbapenem-resistant Enterobacterales, screening, cohort isolation, time to admission, organizational performance

## Abstract

Background: Carbapenem-resistant *Enterobacterales* (CRE) require early identification and appropriate patient placement to prevent in-hospital transmission. Bed Management plays a key organizational role in coordinating screening results and isolation strategies; however, evidence on its impact on patient flow and isolation practices remains limited. Aim: To evaluate whether the implementation of a Bed Management-coordinated structured CRE screening pathway was associated with changes in patient placement appropriateness and time to admission (TTA). Methods: We conducted a retrospective cohort study including all patients with a positive rectal swab for CRE during two study periods (PRE: 2024; POST: 2025) in a tertiary care hospital. A structured CRE screening pathway coordinated by Bed Management was implemented in the POST period. Primary outcomes were cohort isolation rates and TTA. Continuous variables were compared using the Mann–Whitney U test and categorical variables using the chi-square test. Results: A total of 158 CRE-positive patients were included (69 in the PRE period and 89 in the POST period). Patient characteristics were comparable between periods (median age 75 years [IQR 64–81] vs. 73 years [IQR 64–82]; female sex 33.3% vs. 44.9%, *p* = 0.189). Cohort isolation rates were higher in the POST period. Median time to admission (TTA) decreased from 74.8 h (IQR 47.2–124.6) in PRE to 70.7 h (IQR 35.1–139.9) in POST; however, this difference did not reach statistical significance (Mann–Whitney U test, *p* = 0.630). Conclusions: A Bed Management-coordinated CRE screening pathway was associated with improved cohort isolation practices and an observed, non-significant reduction in TTA. These findings suggest that integrating infection prevention workflows with centralized bed allocation may be feasible without adversely affecting admission timeliness. Further studies with larger samples and longer observation periods are warranted.

## 1. Introduction

Screening for carbapenem-resistant Enterobacterales (CRE) includes a set of procedures aimed at the early identification of colonized or infected patients in order to prevent transmission within healthcare settings. The spread of CRE represents a major threat to patient safety, as it is associated with severe infections, limited therapeutic options, and a substantial increase in morbidity, mortality, and healthcare costs [1,2,3,4].

Several studies have demonstrated that the implementation of integrated infection prevention and control strategies, including early screening of high-risk patients, is associated with a significant reduction or, in some settings, interruption of CRE transmission in hospitals [5,6,7,8,9,10]. The combination of admission screening and isolation measures, including cohorting, has also proven effective in high-complexity care environments [8].

Rectal swab screening is the most commonly recommended method for CRE detection and is performed in patients considered at risk, such as hospitalized individuals, those transferred from other healthcare facilities, or patients with previous healthcare exposure [11,12,13,14,15]. Identification of resistant strains is achieved through conventional microbiological techniques and molecular methods, including polymerase chain reaction (PCR), which allows rapid detection of major resistance genes [14,15].

In cases of CRE positivity, international guidelines recommend immediate implementation of containment measures, including single-room isolation, use of personal protective equipment, and rigorous environmental disinfection. When single rooms are unavailable, functional isolation or cohort isolation is recommended, ensuring that patients colonized or infected with different resistance mechanisms are not cohorted together [16,17,18].

In 2022, the World Health Organization emphasized systematic infectious risk assessment as part of standard precautions for infection prevention and control, recommending its implementation at the point of care in all healthcare settings and reassessment when clinical conditions change [17]. At national and European levels, structured surveillance systems have been developed based on early screening and standardized risk-assessment checklists [18,19]. in line with broader WHO recommendations on infection prevention and control programmes [20].

The European Centre for Disease Prevention and Control recently published the third update on carbapenem-resistant Enterobacterales surveillance, reaffirming that active screening, early isolation, and appropriate patient allocation are cornerstone measures for controlling multidrug-resistant organisms (MDROs), according to internationally recognized definitions [12,21]. The document also highlights the importance of structured organizational models and centralized management of care flows to optimize resource use and ensure appropriate infection control practices [12].

At the AOUC Policlinico di Bari, an institutional procedure mandates the use of an anamnestic screening form to guide rectal swab testing in wards not classified as high-risk. This form is completed by admission staff or by the Bed Manager in urgent admissions and included in the patient’s clinical documentation. Leveraging a comprehensive overview of hospital bed availability, the Bed Manager allocates CRE-positive patients to contact isolation in single rooms when available or to cohort isolation according to resistance gene compatibility [7,10,12].

This study aimed to evaluate whether the introduction of this Bed Management-coordinated screening and allocation model resulted in an increase in cohort isolation admissions and to assess its impact on time to admission (TTA).

## 2. Materials and Methods

### 2.1. Study Setting and Screening Procedure

This monocentric retrospective pre–post observational study was conducted in a tertiary-care teaching hospital in Bari (Southern Italy). We compared two consecutive 6-month periods (PRE: 2024; POST: 2025). In both periods, patients presenting to the Emergency Department (ED) underwent an infectious risk assessment at the point of care. When at least one predefined risk factor for carbapenem-resistant Enterobacterales (CRE) colonization was identified, a rectal swab was requested before ward admission and processed by the local microbiology laboratory according to routine microbiological procedures, including culture on selective chromogenic media for carbapenem-resistant Enterobacterales. Identification and antimicrobial susceptibility testing were performed using standard laboratory methods. When indicated, molecular assays were used for the detection of carbapenemase genes. No substantial changes in microbiological screening methods occurred between the PRE and POST periods. The predefined risk factors assessed through the anamnestic screening checklist included: previous CRE colonization or infection, transfer from another healthcare facility, immunosuppression or ongoing chemotherapy, chronic dialysis, presence of invasive devices, incontinence or diarrhoea, presence of ileostomy or colostomy, and uncontained draining wounds. The aim of the screening pathway was to enable early implementation of contact precautions and appropriate bed allocation before inpatient transfer. The turnaround time for microbiological results was determined by routine laboratory workflow and was not modified by the intervention. In general, rectal swab culture results were available within approximately 24–48 h.

### 2.2. Bed Management-Coordinated Allocation Pathway (POST Period)

In the POST period, Bed Management coordinated a structured allocation pathway linking screening information, microbiology results, and real-time bed availability. Specifically, the Bed Manager: (i) verified completion of the anamnestic risk-assessment checklist in the ED; (ii) ensured timely ordering/collection of the rectal swab; (iii) maintained an updated list of patients with pending or confirmed CRE screening; (iv) prioritized placement in single rooms when available; and (v) when single rooms were unavailable, arranged cohort isolation based on compatibility of colonization/infection status and (when available) resistance mechanism, avoiding cohorting of patients with different carbapenemase profiles in line with infection prevention recommendations. This centralized workflow aimed to standardize communication and reduce delays in allocating an appropriate bed while respecting isolation constraints. Operational components of the pathway are summarized in Supplementary Table S1.

**Figure 1:**
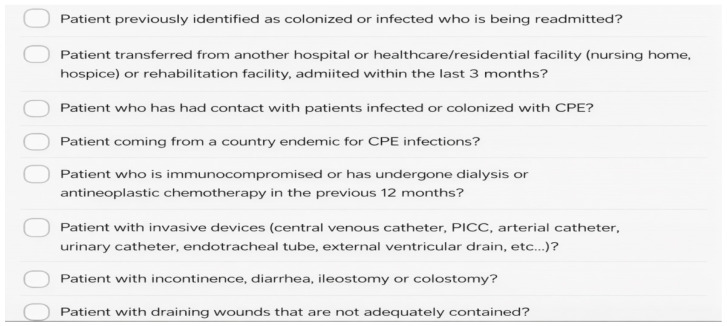
Anamnestic CRE risk-assessment checklist used in the POST period to trigger rectal swab screening in the ED and support early isolation/bed allocation.

**Figure 2:**
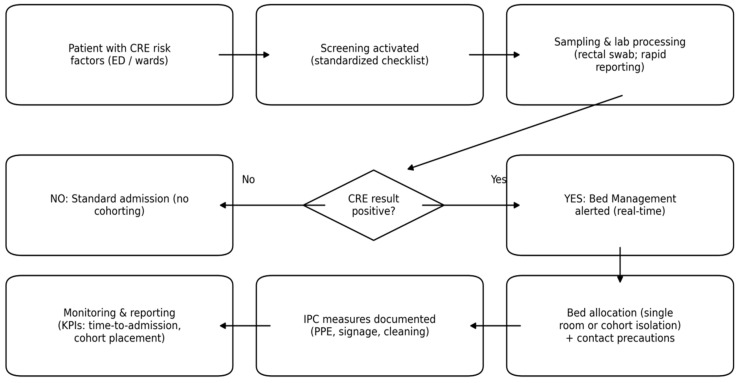
Schematic workflow of the Bed Management-coordinated CRE screening and allocation pathway (POST period).

The anamnestic CRE risk-assessment checklist used to trigger rectal swab screening in the Emergency Department is shown in Figure 1, while the Bed Management-coordinated allocation workflow is illustrated in Figure 2.

### 2.3. Data Sources and Variables

Data were extracted from the hospital information system and the microbiology laboratory reporting system. For each CRE-positive patient identified during the study periods, we collected age, sex, the screening risk factor recorded on the checklist, and bed placement at admission (cohort isolation versus non-cohort placement). Time to admission (TTA) was calculated as the time interval (in hours) between ED arrival time and time of inpatient ward bed allocation. Screening activity denominators (number of patients screened in the ED prior to admission) were also retrieved to describe screening intensity and positivity rates.

### 2.4. Outcomes

The primary organizational outcomes were: (1) the proportion of CRE-positive admissions managed in cohort isolation and (2) TTA (hours). Secondary descriptive indicators included screening volume and positivity rate during each period.

### 2.5. Statistical Analysis

Continuous variables were summarized as median (interquartile range, IQR) and mean ± standard deviation (SD) for descriptive purposes. Group comparisons were performed using the Mann–Whitney U test for continuous variables and the chi-square test (two-sided) for categorical variables. Effect sizes were reported as risk ratios (RR) with 95% confidence intervals (CI) where applicable. Statistical significance was set at *p* < 0.05. Analyses were conducted on de-identified aggregated data.

### 2.6. Ethics

This study was conducted as a retrospective observational analysis of anonymized and aggregated organizational data routinely collected within the hospital information system for operational and quality improvement purposes. According to Italian regulations on retrospective studies using fully anonymized data with no direct patient involvement and no modification of clinical management, formal approval from an Institutional Review Board or Ethics Committee was not required. Therefore, the study was considered exempt from ethics committee approval according to institutional policy.

## 3. Results

### 3.1. Demographic Characteristics

A total of 158 CRE-positive patients were included in the analysis, with 69 patients in the PRE period and 89 patients in the POST period. Patient demographics were comparable between periods. Median age was 75 years (IQR 64–81) in PRE and 73 years (IQR 64–82) in POST (Mann–Whitney U test, *p* ≈ 0.77). Female sex accounted for 33.3% (23/69) of patients in PRE and 44.9% (40/89) in POST, with no statistically significant difference between periods (chi-square test, *p* = 0.189) (Table 1).

### 3.2. Reasons for CRE Screening Positivity

All CRE screening swabs were collected in the Emergency Department before inpatient ward admission. Risk factors leading to CRE screening positivity did not differ significantly between PRE and POST periods. Transfer from other healthcare facilities was the most frequent factor in both groups (Table 2).

### 3.3. Screening Activity and Positivity Rate

During the PRE period, 978 patients underwent CRE rectal swab screening in the Emergency Department prior to ward admission, and 69 tested positive (positivity rate: 7.1%). In the POST period, 1126 patients were screened and 89 tested positive (positivity rate: 7.9%). The difference in positivity rates between periods was not statistically significant (chi-square test, *p* = 0.46; RR = 1.12, 95% CI 0.83–1.52). Screening activity increased by 15.1% in the POST period (1126 vs. 978) (Table 3).

### 3.4. Cohort Isolation

During the POST period, the proportion of CRE-positive patients managed in cohort isolation increased by 4.9 percentage points compared with the PRE period (78.7% vs. 73.9%), corresponding to 19 additional cohort admissions. However, this difference was not statistically significant (chi-square test, *p* = 0.49). The relative likelihood of cohort isolation in the POST period was RR = 1.06 (95% CI 0.89–1.27).

Non-cohort placement (single-room contact isolation or other non-cohort arrangements) accounted for 18/69 (26.1%) admissions in the PRE period and 19/89 (21.3%) in the POST period (Table 4).

### 3.5. TTA (Time to Admission)

Time to admission showed an observed reduction in the post-implementation period. Median TTA decreased from 74.8 h (IQR 47.2–124.6) in the PRE period to 70.7 h (IQR 35.1–139.9) in the POST period; however, this difference was not statistically significant (Mann–Whitney U test, two-sided, *p* = 0.630). Mean TTA showed a similar downward trend, decreasing from 97.6 ± 78.5 h in PRE to 93.3 ± 75.4 h in POST (Table 5).

## 4. Discussion

This study evaluated the organizational impact of a Bed Management-coordinated CRE screening pathway on patient placement and time to admission. The post-implementation period was associated with improved cohort isolation practices, suggesting better alignment between infection prevention requirements and bed allocation processes. Importantly, this organizational improvement was not accompanied by delays in patient admission, as TTA showed an observed reduction that did not reach statistical significance.

Although the reduction in TTA did not reach statistical significance, both median and mean values showed a downward trend following implementation of the structured pathway. These findings suggest that enhanced coordination between infection prevention workflows and centralized bed allocation did not adversely affect patient flow. In a context where isolation requirements are often perceived as a potential barrier to timely admission, the absence of TTA prolongation is a relevant operational finding.

Bed Management may play a key organizational role in translating microbiological screening results into timely and appropriate patient placement decisions. By centralizing information flow and coordinating cohorting strategies, Bed Management can support infection prevention objectives while maintaining operational feasibility. Our findings indicate that such integration is achievable in routine clinical practice without compromising admission timeliness.

The availability of screening denominators allowed differentiation between increased case detection and changes in screening intensity. Although a higher absolute number of CRE-positive patients was observed in the POST period, screening activity increased (1126 vs. 978), while the positivity rate remained comparable (7.9% vs. 7.1%; *p* = 0.46). This finding suggests that the observed increase was largely driven by enhanced screening volume rather than a clear epidemiological surge.

The increase in screening activity observed during the POST period likely reflects improved adherence to the standardized risk-assessment checklist rather than a change in screening criteria. The same screening criteria were applied in both study periods, and the structured Bed Management pathway may have contributed to more consistent checklist completion in the Emergency Department.

From an operational perspective, isolation-capable bed scarcity can contribute to access block and prolonged ED boarding when admitted patients wait for an appropriate bed. Patient flow literature emphasizes that bed allocation, discharge processes, and cross-department coordination are key levers to reduce admission delays and improve hospital-wide throughput [22]. Our findings support the hypothesis that integrating infection prevention constraints into centralized bed allocation is feasible and may mitigate the perceived trade-off between timely admission and appropriate isolation.

Previous reports have shown that dedicated bed management systems and structured patient flow interventions can shorten bed turnover time and reduce boarding without compromising safety [23,24]. These findings are also consistent with process-improvement reports showing reduced time to admit emergency department patients to inpatient beds after targeted operational interventions [25]. In the context of multidrug-resistant organisms, rapid risk assessment and early isolation remain cornerstone measures, but their effectiveness depends on consistent operational execution across the ED-to-ward transition [12,17]. Embedding these steps within Bed Management may therefore represent a pragmatic strategy to translate guideline recommendations into routine practice.

Although the observed improvements did not reach statistical significance, the direction of effect is consistent with the expected mechanism of action: earlier identification of high-risk patients, fewer ad hoc bed moves, and clearer escalation rules when isolation resources are constrained. Future work should evaluate longer observation periods, include ward-level occupancy and crowding indicators, and assess patient-centered outcomes such as length of stay, intrahospital transfers, and incident CRE transmission [10,12].

Finally, this model has potential applications beyond CRE. A similar Bed Management-driven approach could be adapted to other transmission-based precautions (e.g., *Clostridioides difficile*, respiratory viruses) and integrated with electronic alerts and dashboards to support real-time decisions. Such integration could help hospitals balance infection prevention requirements with patient flow during periods of high demand.

## 5. Strengths and Limitations

This study has several limitations. Its retrospective and single-center design limits causal inference and generalizability. The sample size may have been insufficient to detect small differences in TTA, and no predefined non-inferiority margin was established. Additionally, potential confounders such as emergency department crowding, bed availability, and ward-level operational constraints were not formally measured. In addition, no major structural changes in hospital bed capacity, staffing levels, infection prevention policies, or isolation-room availability occurred between the PRE and POST periods. However, the retrospective design did not allow a formal adjustment for operational variables such as emergency department volume or ward occupancy, which could potentially influence time-to-admission metrics. The two study periods were selected to be temporally comparable (consecutive six-month intervals) in order to minimize potential seasonal effects related to infectious disease circulation or hospital demand. Nevertheless, residual temporal confounding cannot be completely excluded. The analysis of time to admission was restricted to CRE-positive patients because the objective was to evaluate how the Bed Management pathway managed patients requiring isolation precautions. Evaluating the impact of the intervention on the entire screened population, including CRE-negative patients, would require a different analytical design focused on overall patient flow and was beyond the scope of the present study. Finally, although screening denominators were available, more granular information (e.g., indication for screening, ward-level screening intensity, and temporal trends) was not available, limiting a more detailed interpretation of changes in CRE detection. Ward-level incidence density of new CRE cases was not analyzed because the focus of the study was on organizational outcomes related to patient placement and admission flow rather than epidemiological surveillance indicators.

Additional limitations include the lack of granular microbiological information (e.g., carbapenemase gene distribution) and the absence of a direct measure of isolation room availability over time. Operational indicators such as hospital bed occupancy rate, emergency department boarding index, or access block metrics were not available in the dataset and were therefore not included in the analysis. Moreover, we did not measure downstream outcomes such as in-hospital CRE transmission, length of stay, or intrahospital transfers, which would provide a more comprehensive assessment of clinical and operational impact. The primary objective of this study was to evaluate the organizational impact of a Bed Management-coordinated screening pathway rather than epidemiological outcomes. Therefore, indicators such as intrahospital CRE transmission, ward-level incidence density, or outbreak occurrence were not included in the study design. These outcomes require microbiological surveillance datasets and longer observation periods and should be explored in future studies evaluating the infection control effectiveness of this organizational model.

## 6. Conclusions

A structured CRE screening pathway coordinated by Bed Management was associated with improved cohort isolation practices and an observed non-significant reduction in TTA. These findings suggest that integrating infection prevention workflows with centralized bed allocation may be feasible without adversely affecting admission timeliness. Larger multicenter studies with longer observation periods and screening denominators are warranted. From an operational standpoint, formalizing the interface between ED screening and bed allocation may support both infection prevention and patient flow, particularly when isolation resources are limited.

## Figures and Tables

**Table 1:** Demographic characteristics of CRE-positive patients (PRE vs. POST).

Variable	PRE (2024, *n* = 69)	POST (2025, *n* = 89)	*p*-Value *
Age, median (IQR), years	75 (64–81)	73 (64–82)	0.77
Age, mean ± SD, years (descriptive)	71.4 ± 15.8	71.9 ± 12.9	—
Female sex, *n* (%)	23 (33.3%)	40 (44.9%)	0.189
Male sex, *n* (%)	46 (66.7%)	49 (55.1%)	—

* Mann–Whitney U test (two-sided) for age; chi-square test for sex.

**Table 2:** Risk factors prompting CRE screening (PRE vs. POST).

Risk Factor	PRE *n* (%)	POST *n* (%)	*p* Value
Previous CRE colonization/infection	1 (1.4%)	2 (2.2%)	0.999
Transfer from another healthcare facility	35 (50.7%)	44 (49.4%)	0.876
Immunosuppression/dialysis/chemotherapy	7 (10.1%)	12 (13.5%)	0.622
Invasive devices	8 (11.6%)	9 (10.1%)	0.812
Incontinence/diarrhoea/ileostomy–colostomy	13 (18.8%)	14 (15.7%)	0.642
Uncontained draining wounds	5 (7.2%)	8 (9.0%)	0.771

**Table 3:** CRE screening activity and positivity rate (PRE vs. POST).

Period	Patients Screened (*n*)	CRE-Positive (*n*)	Positivity Rate (%)	RR (POST vs. PRE), 95% CI	*p*-Value *
PRE	978	69	7.1	Reference	—
POST	1126	89	7.9	1.12 (0.83–1.52)	0.46

* Chi-square test (two-sided).

**Table 4:** Cohort isolation among CRE-positive admissions.

Period	Cohort Isolation Admissions *n* (%)	Δ *n*	Δ %	RR (POST vs. PRE), 95% CI	*p*-Value *
PRE (*n* = 69)	51 (73.9%)	—	—	Reference	—
POST (*n* = 89)	70 (78.7%)	+19	+4.9%	1.06 (0.89–1.27)	0.49

* Chi-square test (two-sided), no continuity correction.

**Table 5:** Time to Admission.

Variable	PRE (2024, *n* = 69)	POST (2025, *n* = 89)	Difference	*p*-Value *
TTA, median (IQR), hours	74.8 (47.2–124.6)	70.7 (35.1–139.9)	−4.1	0.630
TTA, mean ± SD, hours (descriptive)	97.6 ± 78.5	93.3 ± 75.4	−4.3	—

* Mann–Whitney U test (two-sided).

## Data Availability

The data that support the findings of this study are available from the corresponding author upon reasonable request.

## Supplementary Materials

The following supporting information can be downloaded at https://www.mdpi.com/article/10.3390/antibiotics15030290/s1. Supplementary Table S1 reports the core operational components of the Bed Management-coordinated pathway implemented in the POST period.
