# Supplementary material for: Screening and Cohorting of CRE Patients: The Strategic Role of Bed Management in a Monocentric Pre–Post Observational Study
Source: Antibiotics (Basel). 2026 Mar 12;15(3):290. doi: 10.3390/antibiotics15030290 (PMC13024421; doi:10.3390/antibiotics15030290)
Supplement: Supplementary file 1 [file antibiotics-15-00290-s001.zip › antibiotics-4151398-supplementary.pdf]

Supplementary Materials

Supplementary Table S1 reports the core operational components of the Bed Management-coordinated pathway implemented in the POST period.

Supplementary Table S1 - Core components of the Bed Management-coordinated CRE pathway (POST period)

**Component**	**Operational description**	**Primary objective**
Risk assessment checklist	Standardized anamnestic form completed in the ED to identify CRE risk factors triggering rectal swab request.	Early identification of patients requiring contact precautions.
Tracking and communication	Centralized tracking of pending/confirmed CRE screening and structured handover between ED, wards, Bed Management, and IPC team.	Avoid information loss and support timely decisions.
Isolation decision rules	Priority to single-room contact isolation; cohort isolation when needed, with attention to compatibility of resistance mechanisms when available.	Reduce transmission risk while optimizing limited isolation capacity.
Bed allocation and escalation	Real-time bed availability review; escalation to ward coordinators when isolation-capable beds are constrained.	Minimize delays to admission while maintaining IPC requirements.
